# UV-Induced Spectral and Morphological Changes in Bacterial Spores for Inactivation Assessment

**DOI:** 10.1021/acs.jpcb.3c07062

**Published:** 2024-02-07

**Authors:** Rasmus Öberg, Timir B. Sil, Alexandra C. Johansson, Dmitry Malyshev, Lars Landström, Susanne Johansson, Magnus Andersson, Per Ola Andersson

**Affiliations:** † Swedish Defence Research Agency (FOI), Umeå 90621, Sweden; ‡ Department of Physics, Umeå University, Umeå 90736, Sweden; § Swedish Defence Research Agency (FOI), Norra Sorunda 13794, Sweden; ∥ Umeå Centre for Microbial Research (UCMR), Umeå 90736, Sweden

## Abstract

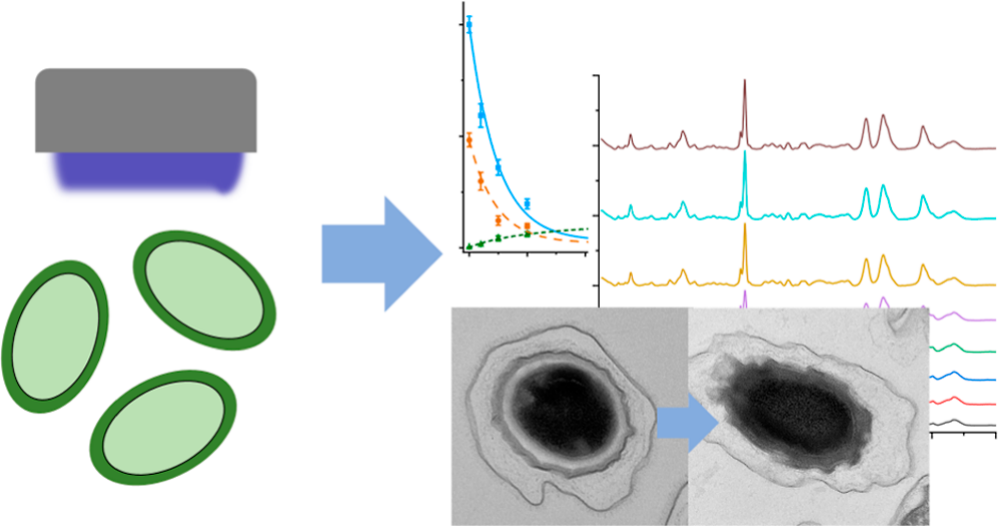

The ability to detect and inactivate spore-forming bacteria is of significance within, for example, industrial, healthcare, and defense sectors. Not only are stringent protocols necessary for the inactivation of spores but robust procedures are also required to detect viable spores after an inactivation assay to evaluate the procedure’s success. UV radiation is a standard procedure to inactivate spores. However, there is limited understanding regarding its impact on spores’ spectral and morphological characteristics. A further insight into these UV-induced changes can significantly improve the design of spore decontamination procedures and verification assays. This work investigates the spectral and morphological changes to *Bacillus thuringiensis* spores after UV exposure. Using absorbance and fluorescence spectroscopy, we observe an exponential decay in the spectral intensity of amino acids and protein structures, as well as a logistic increase in dimerized DPA with increased UV exposure on bulk spore suspensions. Additionally, using micro-Raman spectroscopy, we observe DPA release and protein degradation with increased UV exposure. More specifically, the protein backbone’s 1600–1700 cm^–1^ amide I band decays slower than other amino acid-based structures. Last, using electron microscopy and light scattering measurements, we observe shriveling of the spore bodies with increased UV radiation, alongside the leaking of core content and disruption of proteinaceous coat and exosporium layers. Overall, this work utilized spectroscopy and electron microscopy techniques to gain new understanding of UV-induced spore inactivation relating to spore degradation and CaDPA release. The study also identified spectroscopic indicators that can be used to determine spore viability after inactivation. These findings have practical applications in the development of new spore decontamination and inactivation validation methods.

## Introduction

Several prominent pathogenic bacteria form bacterial spores when exposed to adverse conditions. These include bacteria such as those responsible for food poisoning (*Bacillus cereus*), hospital infections (*Clostridioides difficile*), and deadly diseases such as anthrax (*Bacillus anthracis*).^1,2^ Spore forming bacteria, such as those above, are of particular concern due to the hardiness and resilience of the spore form. Spores are capable of surviving extreme temperatures (>100 °C), disinfection chemicals, as well as ambient conditions for upward thousands of years.^3,4^ Due to their high resilience, many of the decontamination methods that are effective against other pathogens are ineffective against spores, with large discrepancies in decontamination efficiencies even within the same species.^5^ As such, it is important to reliably be able to evaluate a spore sample, even after a decontamination procedure.

One effective method for spore decontamination is ultraviolet (UV) irradiation. Although more effective against vegetative bacteria, UV-C radiation (200–280 nm) has shown to be effective in decontaminating spores on surfaces, as well as in aerosols and suspensions.^6−8^ The mechanisms for UV deactivation have been well explored within bacteria, with the primary mode being UV-induced dimerization of pyrimidine bases in DNA inhibiting transcription and translation, as well as absorption by amino acids tryptophan, tyrosine, and phenylalanine around the range 250–280 nm^9,10^ and at shorter wavelengths <230 nm due to a second absorption band. The mechanism behind UV-induced deactivation of spores is thought to be largely similar to that of vegetative bacteria. However, due to differences in the structure and biochemistry of spores, including a protective chelate of dipicolinic acid (DPA) surrounding the DNA, and an altered DNA configuration, spores can resist between 10 and 50 times the radiation dose at 254 nm as compared to vegetative bacteria.^3,11^

When evaluating a decontamination procedure, and thus the viability of a bacterial spore sample, there are several methods available to employ. The gold standard technique for evaluating spore viability is culture growth on rich medium agar plates,^12,13^ which although reliable is a very time-consuming process, requiring up to 48 h of incubation for spores like *C. difficile*.^14^ As an alternative to this, spectroscopic techniques such as fluorescence and Raman spectroscopies offer a rapid option for following chemical changes in spores, as well as following processes relevant to spore viability, for example, germination.^15−19^ Fluorescence spectroscopy can detect the presence of amino acids like tryptophan and tyrosine, and the fluorescence intensity is a good marker when spore protein structures break down.^15,20^ Similarly, Raman spectroscopy can detect the relative presence of amino acids and indicate the presence of DNA or DPA release.^17,18^ Raman spectroscopy can also utilize deuterium oxide (heavy water) as a marker and follow specific spore-related processes such as germination and outgrowth.^19^

In addition to the spectroscopic methods mentioned above, imaging techniques such as electron microscopy can help resolve the integrity and layer structure of a spore sample. Using scanning electron microscopy (SEM), Zeng et al. showed that bacterial spores exposed to disinfection chemicals and UV radiation, to some extent, have surface damage or appear wrinkled.^21^ Also, transmission electron microscopy (TEM) has been widely used to evaluate changes in spore layer structures. This includes changes such as core damage or shifting in the spore membrane and coat structures.^18,22^ These spectroscopic and imaging techniques can individually tell a lot about the change in spore composition during UV-induced inactivation; however, using them in tandem, we can more clearly elucidate the sequence of events involved in the inactivation procedure.

In this work, we utilize spectroscopic and imaging techniques to evaluate changes in *Bacillus thuringiensis* (*B. thuringiensis*) bacterial spores after exposure to UV radiation. Using fluorescence spectroscopy, we assess the spore protein structure by examining changes in individual aromatic amino acids. Additionally, we examine variations in the fluorescence emission from DPA following exposure to UV light. To observe the chemical composition and changes in individual spores as a result of UV radiation, we use Raman spectroscopy. Morphological changes on spores from UV radiation are evaluated using SEM and TEM imaging. The results of this work offer new understanding of the mechanisms behind UV-induced spore decontamination relating to protein degradation and CaDPA release, alongside novel information about spectroscopic benchmarks and morphological indicators associated with spore inactivation.

## Methods

### Spore Preparation and Decontamination

*B. thuringiensis* ATCC 35646 cells were grown on BBL AK 2 agar plates (210912, BD Biosciences) and incubated overnight at 30 °C. These cells were then scraped off the agar and transferred to a 1.5 mL Eppendorf tube, in which they were centrifuged to remove leftover growth media. The cells were then left to sporulate by storing them at 4 °C overnight. To purify the resulting spore suspension, we centrifuged them in deionized water at 5000 *g*, for 5 min 5 times, discarding the supernatant and resuspending the pellet each time. After purification, we resuspended the spores in deionized water and stored them at 4 °C. To confirm our results were not affected by biological debris, we performed measurements on spores that had undergone further purification. Spore suspensions were layered on top of a 50% nonionic density gradient (Histodenz, D2158, Sigma-Aldrich) and were centrifuged at 5000 *g* for 10 min.^23^ The supernatant was then discarded, and the purified spores were washed three times in deionized water to remove the dissolved Histodenz.

We deactivated spore suspensions at concentration 10^6^ spores/mL by exposing them to UV radiation from an Hg lamp (EVG65-80W/1,5A-PH, ZED). The spore suspension was placed in 3 mL quartz glass cuvettes (10 mm, Agilent) transmitting UV radiation within the full spectrum of the Hg lamp. The cuvettes were placed under the Hg lamp at a distance of 20 cm from the lamp, exposing the sample to UV radiation at a power of 100 mW for periods of time ranging from 5 s to 1 h. To evaluate decontamination, we successively diluted a 100 μL aliquot of spore suspension, distributed it on blood agar base plates, and incubated at 27 °C for 24 h. After incubation, the plates were photographed and the number of colonies counted as described by Johansson et al.^13^

### Nuclear Magnetic Resonance Spectroscopy

^1^H nuclear magnetic resonance (NMR) spectra were recorded using a Bruker Avance III Ultrashield 500 MHz NMR spectrometer equipped with a Cryoprobe 5 mm BBFO head at 500 MHz at 296 K. Chemical shifts are referenced to solutions in deuterium oxide (D_2_O) [residual HOD (δ_H_ 4.79 ppm), as internal standards]. The initial DPA concentrations were 100 mM in D_2_O.

### Absorbance and Fluorescence Spectroscopies

To measure absorbance from the spore suspensions, we used a UV–vis spectrophotometer (Lambda 650, PerkinElmer). We measured the absorbance on 10^6^ spores/mL suspensions between 210 and 800 nm, with a step size of 1 nm, and excitation and emission slit widths were 5 nm. A deionized water sample was used for reference, and the resulting absorbance spectra were corrected to account for Rayleigh and Mie scattering. Shown absorbance spectra are averaged from three technical replicates.

To measure steady-state fluorescence, we used a fluorescence spectrophotometer (Cary Eclipse, Agilent) with a built-in magnetic oscillator to allow stirring during fluorescence measurements. The spectrometer contained two single monochromators, one each for excitation and emitted light, and a slit width of 5 nm was used. The suspensions were then placed in 3 mL, 10 mm quartz cuvettes. We excited the 10^6^ spores/mL suspensions between 210 and 500 nm with a step size of 10 nm, and the fluorescence emission was measured between 220 and 800 nm with a step size of 1 nm. Shown fluorescence spectra are excitation–emission matrices (EEMs) averaged from three technical replicates. Curve fits on absorbance and steady-state fluorescence evolutions were produced in Origin (2020, OriginLab), using exponential decay models *f*(*t*) = *a* e^–*t*/τ^ + *y*_0_ and sigmoidal logistic models .

To measure the fluorescence decay times, we used time-correlated single-photon counting (TCSPC). We used a pulsed nanoLED (NanoLED-290, Horiba Scientific) emitting at 285 nm, with fluorescent light at 330 nm collected using a picosecond photon detector (PPD-850, Horiba Scientific). Acquired TCSPC data was analyzed using the IBH DAS6 analysis software (IBH Decay analysis v6.1.51, Horiba Scientific) and Origin and fit to three-component exponential decay models , where τ_*x*_ are the fluorescence lifetimes of the fluorescing species, and *a*_*x*_ are the respective proportionality constants, corresponding to the relative amplitude of the fluorescing species. A triple decay fit was chosen due to the complexity of the sample, with several fluorescing agents.

### Raman Spectroscopy

To acquire Raman spectra from single spores, we used a custom design laser tweezer Raman spectroscopy setup, as described in greater detail in previous works.^24,25^ The system is constructed around an inverted microscope (IX71, Olympus), with a 785 nm wavelength laser (08-NLD, Cobolt) with an output power of 120 mW. The beam has a Gaussian profile and is coupled into the microscope system using a dichroic short-pass mirror with a cutoff wavelength of 650 nm (DMSP650, Thorlabs).

To focus the beam onto a sample, we use a 60× water immersion objective with a working distance of 0.28 mm and numerical aperture 1.2 (UPlanSApo, Olympus). The backscattered Raman light is then collected through the microscope objective, after which the Rayleigh scattered light is filtered out using a 785 nm notch filter (NF785-33, Thorlabs), and further filtered using a 150 μm diameter pinhole in the telescope’s focal point. The filtered light is then coupled into a spectrometer (model 207, McPherson), with the Raman scattered light dispersed through a 600 grooves/mm, 800 nm blaze, holographic grating. Finally, Raman spectra are captured using a Peltier-cooled CCD camera (Newton 920N-BR-DDXW-RECR, Andor), working at −95 °C. The spectral resolution of our instrument is <3 cm^–1^, and to control the detector and acquire Raman spectra, we use the Solis (Solis v4.30, Andor) software.

To prepare a sample for Raman spectroscopy, we drop 10 μL of the 10^6^ spores/mL spore suspension onto a 60 × 24 mm glass coverslip (no. 1, Paul Marienfeld GmbH & Co., Lauda-Königshofen, Germany). We then place a vacuum grease ring (Dow Corning, Midland, MI) around the drop and seal the sample using a 20 × 20 mm coverslip (no. 1, Paul Marienfeld GmbH & Co., Lauda-Königshofen, Germany). Raman spectra were recorded between 600 and 2000 cm^–1^ through 10 accumulations of 10 s each with a laser power of 60 mW at the sample. The total applied energy by the laser onto the spore is approximately ∼6 J, well below the 20 J shown to be harmful to spores.^26^ For each exposure time, we acquired spectra from 30 separate spores, with shown spectra averaged from these 30 measurements. For background correction of the spectra, we captured spectra of the suspension. To maintain a uniform baseline with minimal interference from the coverslip, we conducted Raman signal measurements at a fixed distance of 100 μm above the glass surface.

To baseline correct our Raman spectra, we used an asymmetrical least-squares algorithm^27^ with λ = 10^5^ and *p* = 10^–3^. We smoothed the spectra using a first-degree Savitzky–Golay filter (frame length of 5). These functions are part of an open-source Matlab program provided by the Vibrational Spectroscopy Core Facility.^28^ To see how the peak intensities changed over time, we normalized the spectra with respect to their initial values.

To perform statistical analysis of differences in the CaDPA peak intensities depending on peak location and time, we used Prism (Prism 9.3, GraphPad Software). Prism was also used to perform a two-way ANOVA with Dunnett’s multiple comparisons test, comparing peak heights at different exposure times to the starting (0 min) time point. Graphs were plotted in Origin. Curve fits for the data were taken in Origin for the mean peak height values with the standard deviation as weighing data. Full parameter fits of Raman data can be found in the Supporting Information.

### Light Scattering Measurements

To measure light scattering from UV-exposed spore suspensions, we used a flow cytometer (Accuri C6, BD), equipped with the BD CSampler hardware and software. Ahead of measurements, spore suspensions were diluted 10-fold to not overcrowd the cytometer. 100 μL of spore suspension were processed for each sample at a flow rate of 28 μL/min. To optimize the system to record interactions with spores at ∼1 μm, we set the recording threshold to 40 000. We then recorded forward scattering events with three technical replicates for each exposure time. Curve fits for the number of scattering events were produced in Origin, using an exponential decay model *f*(*t*) = *a* e^–*t*/τ^ + *y*_0_.

### Electron Microscopy

We generated SEM images from 10 μL spore suspension drops and air-dried on a glass slide. Ahead of imaging, we coated the sample with a ∼5 nm layer of platinum using a Quorum Q150T-ES sputter coater. We then imaged spores using a Carl Zeiss Merlin FESEM electron microscope using the InLens imaging mode at magnifications of 7500–50 000×.

To prepare spores for TEM, we fixed the spore suspensions using 2.5% glutaraldehyde (TAAB Laboratories, Aldermaston, England) in 0.1 M PHEM buffer and then postfixed them in 1% aqueous osmium tetroxide. To further dehydrate the spores, we washed them in ethanol and acetone, after which they were embedded in Spurr’s resin (TAAB Laboratories, Aldermaston, England). The 70 nm spore sections were then post-contrasted in uranyl acetate and Reynolds lead citrate ahead of imaging. We imaged the spores using a Talos L120C (FEI, Eindhoven, The Netherlands) operating at 120 kV. Micrographs were acquired using a Ceta 16M CCD camera (FEI, Eindhoven, The Netherlands), equipped with TEM Image and Analysis software ver. 4.17 (FEI, Eindhoven, The Netherlands).

## Results and Discussion

### UV-Exposed Spores Show Changes in Absorbance and Fluorescence Spectra Relating to Amino Acids and DPA

**Figure 1:**
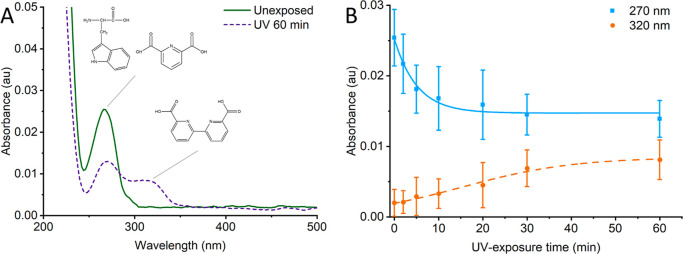
Change in absorbance for spores exposed to UV radiation. Spores exposed to UV (purple dashed) show a decrease in the absorbance band around 270 nm compared to their nonexposed counterparts (green solid) while exhibiting an increase in absorbance around 320 nm. These two changes are attributed to the breakdown of amino acids and the dimerization of DPA, respectively (A). Curves show the intensity of the 270 nm (blue solid) and 320 nm (orange dashed) peaks for samples exposed to different UV exposure times (B). The data sets are fitted to an exponential decay function with fitting parameters *y*_0_ = 0.0147, *a* = 0.0113, and τ = 5.113 (*R*^2^ = 0.98) and a sigmoidal logistic growth function with fitting parameters *a* = 0.00845, *t*_c_ = 15.25, and *k* = 0.0792 (*R*^2^ = 0.98), respectively.

To measure the UV-induced spectral and morphological changes in *B. thuringiensis* spores, we exposed 10^6^ spores/mL suspensions to UV radiation from an unfiltered Hg lamp with an emission profile as can be seen in Figure S1. The Hg lamp has the most prominent line at 253.7 nm; thus, spores are exposed mainly to UV-C. After exposing the spores to UV light, we measured the absorbance of the spores between 220 and 800 nm, see Figure 1A, with no measurable absorbance above 400 nm for the studied spore concentration. Unexposed spores show a single clear absorbance peak around 270 nm. This peak has previously been attributed to several common biochemical compounds, including tryptophan, tyrosine, NAD+/NADH, and to a lesser degree phenylalanine.^29,30^ Furthermore, measurements on pure DPA solution show a significant contribution from DPA to this absorbance peak (Figure S2). After the spores have been exposed to UV radiation between 5 and 30 min, this 270 nm peak gradually decreases (Figure 1B), suggesting a decrease in the concentration of one or several of the biochemical compounds due to UV-induced chemical breakdown. Specifically, amino acids such as tryptophan and tyrosine are known to be sensitive to UV radiation, inducing the synthesis of photoderivatives and free radicals as described by Creed.^31,32^ As can be seen in Figure 1A, we also notice the appearance of a new absorbance peak from the spore sample at 320 nm following UV exposure. We attribute this to the dimerization of DPA as described by Nardi et al.^33^ This matches well with our NMR-spectroscopy measurements on DPA and its photoproducts as measured upon UV irradiation (full data in the Supporting Information and Figure S3). It should be noted that the NMR spectra presented by Nardi et al. are those from the esterified versions of the bipyridinic photoproduct, while the photoproduct presented herein likely corresponds to the nonesterified version with free carboxylic acid groups. The photoactivity of DPA has been previously observed, where studies indicate that UV radiation triggers a reaction between DNA and photosensitizing DPA, reducing spore viability.^34^ From this, it follows that the increase and subsequent stagnation in absorbance are caused by the spore DPA content having dimerized and undergone photolysis with DNA.

**Figure 2:**
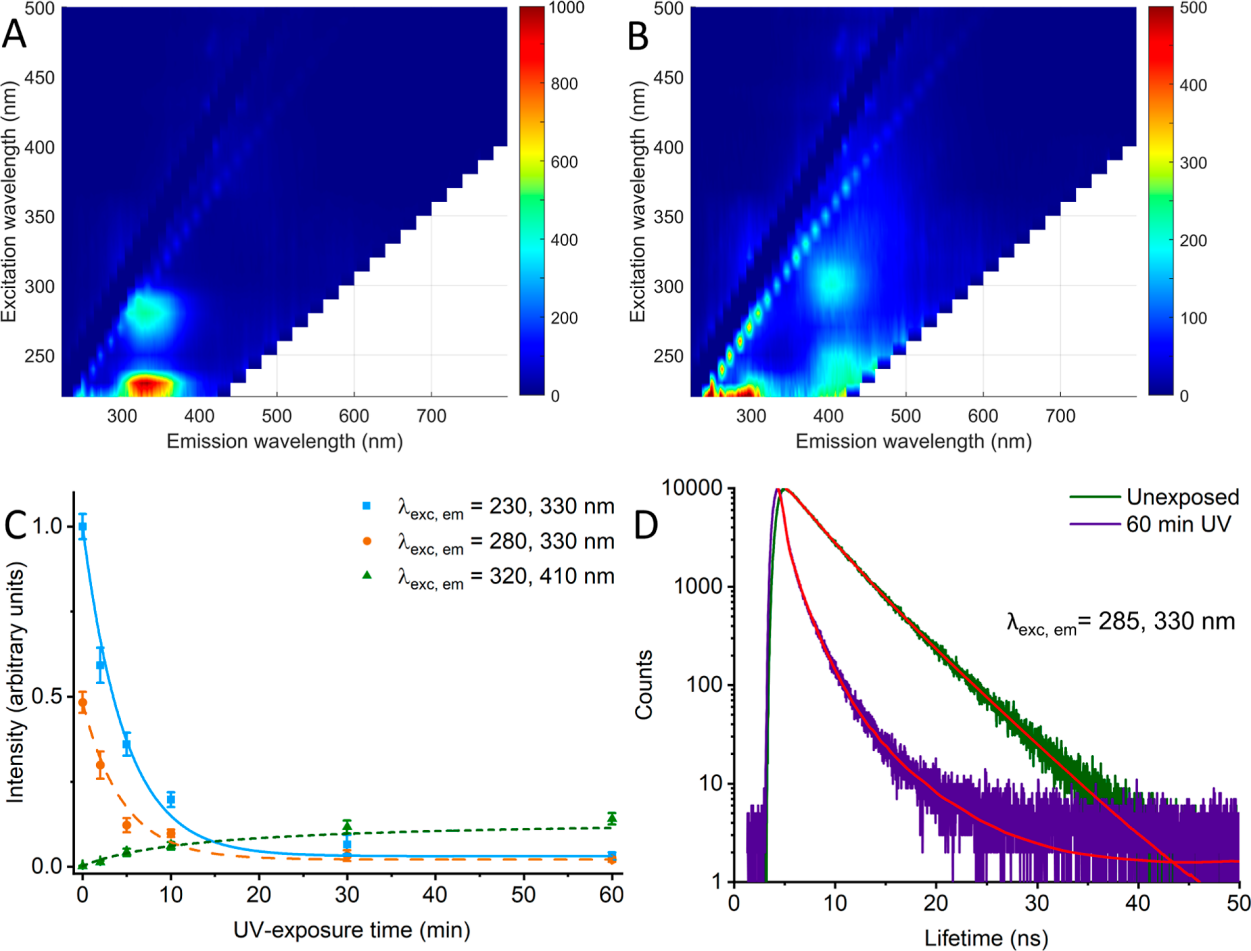
2D fluorescence EEM for unexposed spores and spores exposed to UV radiation. Unexposed spores display two clear fluorescence peaks at λ_exc,em_ = 230, 330 nm and λ_exc,em_ = 280, 330 nm (A). These peaks are largely absent after 60 min UV exposure, while a new peak appears at λ_exc,em_ = 320, 410 nm (B). Notice the difference in scale between (A) and (B). These two former peaks follow an exponential decay (blue/orange dashed) with fitting parameters *y*_0_ = 0.0321, *a* = 0.996, and τ = 4.341 (*R*^2^ = 0.99) and *y*_0_ = 0.0155, *a* = 0.490, and τ = 3.579 (*R*^2^ = 0.98), respectively, while the latter follows a sigmoidal logistic increase curve (green short dashed) with fitting parameters *a* = 0.130, *t*_c_ = 10.27, and *k* = 0.222 (*R*^2^ = 0.97) (C). λ_exc,em_ = 285, 330 nm fluorescence emission follows a triple exponential decay fit with lifetimes around 4.59, 2.37, and 0.56 ns (green), which shortens drastically with increased exposure (purple). Red lines show respective decay fit (D).

Next, we measured the fluorescence spectra from UV-exposed spores. EEM for unexposed spores and spores exposed to 60 min of UV radiation can be seen in Figure 2A,B. Unexposed spores display two clear fluorescence peaks at λ_exc,em_ = 230, 330 nm and 280, 330 nm, respectively. These results fit previous measurements of free molecular tryptophan and tyrosine in water solution well.^29^ Phenylalanine also displays fluorescence with a similar profile as tyrosine; however, due to its lower quantum yield, it is largely indistinguishable in the spore spectra. The fluorescence from these species dissipates quickly after UV exposure, as can be seen in Figure 2C, with the fluorescence intensity of both peaks decreasing to less than 20% of their original intensity after 10 min of exposure. It should be noted that spores are largely inactivated (∼log 4 reduction) already after 2 min of exposure (Figure S4); however, these effects at longer exposure times may be relevant for indicating complete inactivation and associated chemical changes. Repeating these fluorescence measurements on a spore suspension purified through Histodenz, we observe similar spectral characteristics, see Figure S5. In addition to ensuring that the spectral signal originates from a pure sample, this also suggests that a less rigorous cleaning procedure may be acceptable in measurements on live samples for inactivation validation purposes. Looking at the fluorescence lifetime of the emitting residues using time-correlated single-photon counting (TCSPC), we find at λ_exc,em_ = 285, 330 nm the fluorescence emission fits well to a triple fluorescence decay profile (χ^2^ = 0.92), as can be seen in Figure 2D. Such a fit correlates with fluorescence lifetimes of 4.59, 2.37, and 0.56 ns (relative amplitudes 4.69, 40.45, and 54.86), with the former two corresponding well to recorded time constants for tyrosine and tryptophan within protein structures.^35,36^ After 60 min of UV exposure to the spores, these components with longer fluorescence lifetimes disappear, with resulting recorded lifetimes (τ) 0.93, 0.04, and 0.52 ns, all <1 ns (relative amplitudes 15.73, 81.10, and 3.16). The shorter lifetime <100 ps (lower than time resolution of the TCSPC system) likely corresponds to background scattering and emission from short-lived excited states of breakdown products.

After 30–60 min of exposure, we observe an increase and then stagnation in the steady-state fluorescence peak at λ_exc,em_ = 320, 410 nm. Fluorescence in this area has been previously reported from pure DPA degrading into photoproducts, with significant fluorescence observed after UV exposure,^37^ or when observing high concentrations of DPA.^38^ The study by Malyshev et al. using high concentrations of DPA suggests that the breakdown of DPA into the observed fluorescent photoproducts may not be induced but rather catalyzed by UV radiation. These observations were made on a pristine DPA solution in water, and such breakdown will not necessarily be observed in spores due to the glassy composition of water and DPA in the spore core, as well as the chelated nature of the DPA.^39,40^ We further confirm this λ_exc,em_ = 320, 410 nm to be the result of UV-induced DPA photoproduct (Figure S6). Thus, we conclude that absorbance and fluorescence spectroscopies show structural degradation of fluorescent amino acid structures in spore suspensions described by exponential decay over increased UV exposure, alongside a slower logistic increase in dimerized DPA.

### Raman Spectroscopy Reveals Rapid DPA Release and Slow Protein Degradation in UV-Exposed Spores

Micro-Raman spectroscopy is a sensitive method to study chemical changes in individual spores. We, therefore, utilized this technique to observe how UV radiation changed the biochemical composition of spores. Of particular interest is the spore’s release of DPA since that is a good indication if a spore body is damaged. Once initiated, DPA release into water is completed in less than 1 min.^26,41^ However, other changes, such as damage to the spore protein structure, can also be measured. These changes are particularly interesting in the context of the resilience of spore chemical components to UV.

**Figure 3:**
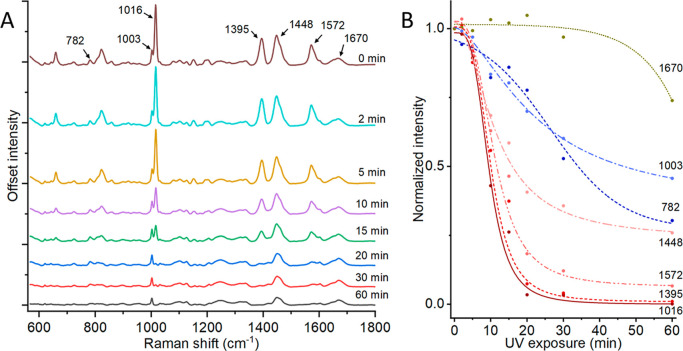
Averaged Raman spectra of spores with different UV exposure times (A). Each spectrum is a composite of two spectra from the ranges 600–1400 and 1200–1950 cm^–1^ for a total of *n* = 60 spores for each spectrum. Normalized intensity of selected peaks over time with fitted curves, demonstrating the different decay profiles of different spore components (B).

The major peaks of bacterial spores have previously been described, and we found the Raman spectra of untreated spores in this work to align with the literature.^39,42,43^ The average spectra of spores in the 600–1950 cm^–1^ range are shown in Figure 3A. The major Raman peaks at 660, 825, 1016, 1395, and 1572 cm^–1^ stem from spore’s calcium dipicolinic acid (CaDPA) content. Other important peaks are the spore DNA peak (782 cm^–1^), phenylalanine as an indicator of protein content (1003 cm^–1^), and the amide I band^44^ in the 1600–1700 cm^–1^ region. The 1450 cm^–1^ region is a reported location for both CaDPA and δ CH_2_,^45^ both of which are present in the spore. Peak assignments are summarized in Table 1.

**Table 1:** Peak Locations and Assignments of Prominent Spore Raman Peaks

peak location (cm^–1^)	assignment
660	CaDPA
782	DNA O–P–O stretch
825	CaDPA
1003	phenylalanine ring breathing
1016	CaDPA ring breathing
1395	CaDPA
1448	δ CH_2_ and CaDPA
1572	CaDPA
1600–1700	amide I (protein backbone)

UV radiation is destructive and degrades both protein and DNA, as well as causing dimerization of DPA. To follow these changes over time, we tracked the evolution of the Raman spectra over 1 h of UV exposure at different time points. As shown in Figure 3A, different peaks decrease in intensity at different rates. The Raman peaks related to CaDPA decreased quickly after 10 min of UV exposure, consistent with previous studies on CaDPA release. The decay of other peaks was slower. We tracked the evolution in the peak intensity of these peaks in detail, see Figures 3B and S7. We found several different decay profiles for spore peaks. The peaks 1016, 1395, and 1572 cm^–1^ have a similar logistic decay profile. The majority of spores lose these peaks in the period between 10 and 15 min, and by 20 min the intensity of these peaks has largely fallen down to noise levels. Based on our observations, we hypothesize that the release of CaDPA from damaged spores is the cause. We also note that the phenylalanine peak at 1003 cm^–1^ decays slower. Even after 1 h of UV exposure, around 50% of the initial peak intensity remains. This is noteworthy as fluorescence from similar amino acids, tryptophan and tyrosine, both decay by over 90% after only 20 min. It is worth mentioning that tryptophan showcases a Raman peak around 1000 cm^–1^, which bears resemblance to the phenylalanine peak, albeit with lesser recognition. This suggests that the differences in observed degradation rates between fluorescence and Raman spectral signatures are more likely to be attributed to differences in molecular mechanics rather than discrepancies in phenylalanine’s ability to resist UV radiation when compared to other amino acids.^46^

Damage to DNA in the spore was observed by the loss in intensity of the 782 cm^–1^ peak (the O–P–O stretch in DNA) over the 60 min exposure time (Figure 3B). The decay profile exhibited a slow decay, with around 70% of the initial peak intensity diminishing in 60 min (Figure S7). However, it is necessary to explore further to determine the accuracy of this curve. This is due to the variability present in the data and the low initial peak value. Further, it is unlikely for UV radiation to directly break the O–P–O DNA backbone of inactive DNA.^47^ Thus, the decay in peak intensity may be a combination of disruption of DNA in the core and loss of DNA fragments attached to the outer layers of the spore.

The 1448 cm^–1^ peak follows a decay profile with two time constants. After a rapid decay within the first 10–15 min similar to other CaDPA peaks, we observe a slower decay, with the peak decaying to ∼35% of its initial intensity after 1 h UV exposure. Therefore, we suggest that the 1430–1470 cm^–1^ region consists of two peaks that overlap. One of these is CaDPA, and the other is a CH_2_ peak (lipid or protein). We could indeed fit a curve for the 1448 cm^–1^ as a 60:40 weight combination of the fitted curves at 1016 and 1003 cm^–1^, respectively.

Finally, our findings indicate that the 1670 cm^–1^ band exhibited the highest UV tolerance, as its intensity remained relatively stable for the initial 30 min and only experienced a 25% decrease after 60 min of illumination. Note that the 1670 cm^–1^ band is in the amide I region and thus corresponds to the polypeptide backbone in the spore proteins. This part of the protein is known for its exceptional compositional stability, making it likely that it also has a certain level of resilience against UV radiation, especially when contrasted with the more chemically unstable benzene ring structure found in phenylalanine (1003 cm^–1^). Overall, since the chemical structure and spectroscopic fingerprint are mainly similar between spore species, we expect these results to be largely translatable to other spore species both within and outside the *Bacillus* genus.^43,48^ One should, however, observe that the exposure time to notice specific spectral changes may differ depending on, among other things, the size of the spore. Full parameter fits of Raman data can be found in the Supporting Information.

To pinpoint the effects of UV light on the Raman fingerprint of DPA alone, we exposed aqueous 30 mM DPA for 60 min in the same conditions as the spores. We used DPA, as the CaDPA chelate is much less stable in dilute aqueous solution lacking buffers, while our focus was directed toward the effects within the covalent bonds of the molecule. DPA was previously reported to dimerize in the presence of UV, but the changes in the Raman spectra have not been previously documented.

The Raman spectra of the pure DPA change greatly with increased exposure, indicative of a structure change as previously shown using NMR (Figure S3). As shown in Figure S8, several peaks of DPA at 60 min have disappeared, while other peaks appeared. The remaining peaks, such as the 1572 cm^–1^ peak, have also shifted. Furthermore, microscopy images indicate that the crystal shape of DPA changes drastically after UV exposure (Figure S9). Pure DPA forms long fibrous crystals, but this characteristic is lost in UV-treated DPA, which instead forms irregular structures without a consistent shape. Thus, these results show that UV illumination induces structural and chemical changes in DPA.

### SEM/TEM and Light Scattering Measurements Show Shriveling and Core Leakage of UV-Exposed Spore Body

**Figure 4:**
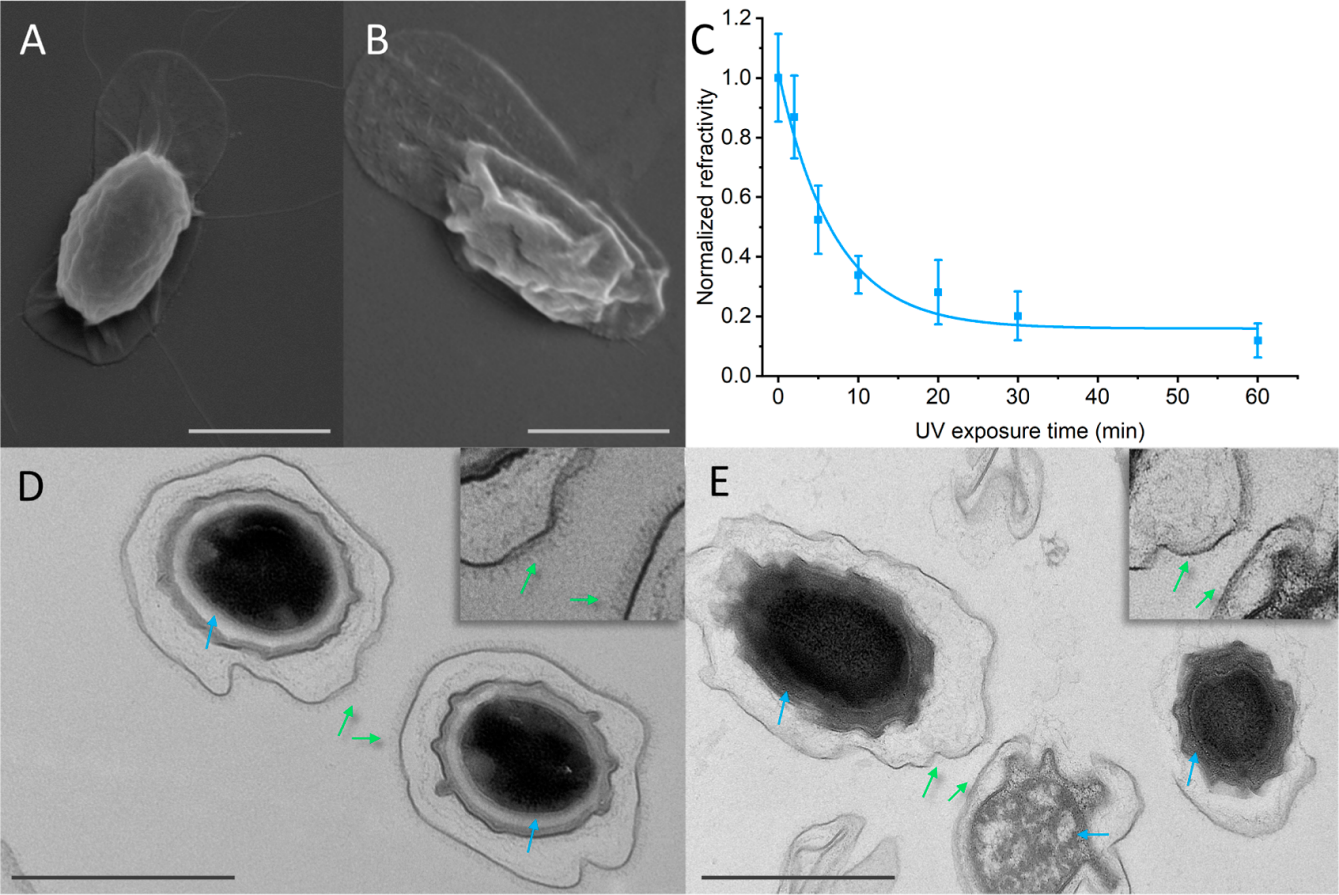
Directly observed changes in spore morphologies from UV exposure. Using SEM, unexposed spores appear plump, showing no sign of spore collapse (A), while spores exposed to 60 min of UV irradiation generally appear more shriveled (B). This is further reflected by an exponential decrease in number of light scattering spores over increased UV exposure time, as fitted with parameters *y*_0_ = 0.175, *a* = 0.881, and τ = 6.815 (*R*^2^ = 0.97) (C). From TEM images, the internal structure of the untreated spores (D) demonstrate a clear separation between spores’ inner layers: the coat (dark ring), cortex (white ring, blue arrows), and core (central dark oval). Spores also have a clearly identifiable nap around spores (green arrows, figure and inset). After 60 min illumination (E), there is leakage of material from the core into the cortex, and the spore nap is no longer visible. Scale bars are 1 μm.

To assess spore morphology and integrity, we used electron microscopy. We used SEM to assess the spore surface and integrity and TEM to check for internal disruption. Spores imaged with SEM (Figure 4A,B) looked markedly different after 1 h of UV exposure. Compared to the untreated spores, UV-exposed spores appeared collapsed and wrinkly, as previously observed by Zeng et al.^21^ In addition, we observed more debris that could not be clearly identified but was present in the UV-treated sample, likely originating from spore fragments (Figures S10 and S11). This observation of collapsed spores was further strengthened by measuring the number of light-scattering entities above a threshold of UV-exposed spore dispersions. We found that increased UV exposure results in a lower count of visible objects of ∼1 μm size (Figure 4C). The number of light-scattering objects gradually decreases following an exponential decay fit, suggesting that the spore integrity is compromised even at relatively low exposure times around 5–10 min, with more resilient spores collapsing after longer exposure.

By imaging the spores using TEM, we can observe changes to the spore layer structure. While there were damaged spores in all samples, likely due in part to the harsh TEM preparation procedure, we see numerous spores with intact layer integrity in the sample that was not UV-treated (Figures 4D and S12). These spores have clearly visible individual layers, from the spore core, cortex, spore coat, as well as the membrane appearing intact. Furthermore, the outer spore is covered by a nap. However, after 1 h of UV exposure, we found significant changes in the layer structure in the spores (Figures 4E and S13). We did not find any fully intact spores in the sample. Most prominently, we found the core content leaked into the cortex, as shown by a significantly darker, electron-dense area between the core and the initial spore coat. This indicates a release of DPA/DNA content otherwise contained in the spore core. We further found that the proteinaceous layers of the spores (coat and exosporium) lost most of their integrity, appearing thinner and less well defined in TEM images, and the nap is no longer present. This coincides well with the previous spectroscopic observation of a breakdown in the amino acids, making up the protein structures. We therefore conclude that we observe a shriveling of the spore structure with increased UV exposure, and leakage of spore core contents and disruption of proteinaceous coat and exosporium layers.

## Conclusions

Due to spore-forming pathogenic bacteria, robust decontamination and viability protocols are vital in industrial, healthcare, and defense sectors. In this work, we investigate how decontamination using UV radiation affects spores’ chemical structure and morphology, features that are important in many detection assays. Using absorbance and fluorescence spectroscopies, we show that fluorescent amino acid structures in spore suspensions quickly deteriorate with increased UV exposure, with the fluorescence signal at λ_exc,em_ = 230, 330/280, 330 nm shrinking by ∼80% after 10 min of exposure. Over the same time span, we show the emergence of a fluorescence peak from dimerized DPA at λ_exc,em_ = 320, 410 nm. This suggests a rupturing of the proteinaceous spore layers and subsequent photoreactions of leaked spore core content. Furthermore, laser tweezer Raman spectroscopy analysis shows rapid spore DPA release in spores within 10–20 min of UV exposure as shown in bands at, for example, 1016 and 1395 cm^–1^, with slower and more gradual disruptions of protein structures, especially the protein backbone represented by the amide I band around 1670 cm^–1^. SEM and TEM imaging of spores suggest spore collapse and the breakdown of proteinaceous layers and leaking of core contents. Thus, research provides novel and multimodal insight into UV-induced spore inactivation, suggesting that UV radiation causes a breakdown in spore protein structures, with subsequent DPA release and dimerization. Changes in spectral responses for DPA release and dimerization may further prove useful in developing spore inactivation procedures, as indicators in confirming spore inactivation post UV decontamination.

## Supporting Information Available

Additional information on DPA and photoproduct NMR data, as well as fitting information for Raman evolution curves, emission profile from Hg lamp, spore inactivation curve, and additional spectral and morphological descriptions of spores and DPA (PDF)
