# Supplementary material for: UV-Induced Spectral and Morphological Changes in Bacterial Spores for Inactivation Assessment
Source: J Phys Chem B. 2024 Feb 7;128(7):1638–46. doi: 10.1021/acs.jpcb.3c07062 (PMC10895659; doi:10.1021/acs.jpcb.3c07062)
Supplement: Supplementary file 1 — jp3c07062_si_001.pdf [file jp3c07062_si_001.pdf]

Supporting Information for Publication

UV-induced Spectral and Morphological Changes in Bacterial Spores for Inactivation Assessment

Rasmus Öberg[a,b], Timir B. Sil[b], Alexandra C. Johansson[a], Dmitry Malyshev[b], Lars Landström[c], Susanne Johansson[a], Magnus Andersson[b,d*], Per Ola Andersson[a,*]

[a] Swedish Defence Research Agency (FOI), Umeå, 90621, Sweden
[b] Department of Physics, Umeå University, Umeå, 90736, Sweden
[c] Swedish Defence Research Agency (FOI), Norra Sorunda, 13794, Sweden
[d] Umeå Centre for Microbial Research (UCMR), Umeå, 90736, Sweden

*Corresponding authors

Contents

Photoreaction of DPA monitored by ^{1}H NMR 2

Parameter values for fitted curves in Figure 3B 3

Supplementary figures 4

References 17

Photoreaction of DPA monitored by ^{1}H NMR

The photoreaction of DPA was monitored by ^{1}H NMR spectroscopy at 1, 2 and 6 hours of irradiation. ^{1}H NMR spectra collected at each time point show the decrease of DPA and the formation of photoproducts over time (Figure S3). The ratio of integrals for ^{1}H NMR signals of the protons of DPA and the main photoproduct was used to determine the remaining amount of DPA at 1 h and 2 h to be 85 % and 45 % respectively. After 6 h irradiation most ^{1}H NMR signals are lost, neither DPA nor the photoproduct obtained in the 1 h and 2 h samples can be observed, this is most likely due to formation of insoluble polymers. The main photoproduct observed at 1 h and 2 h irradiation is assumed to be 2,2´- Bipyridine-6,6-dicarboxylic acid formed by decarboxylative photodimerization. This agrees with the observed ^{1}H NMR signals and a previous spectroscopic study of DPA by Nardi et al. [1] who produced the esterized dimer molecule observed in this work. It should however be noted that the specific photoproduct has not been isolated and characterized due to the low solubility of 2,2´-Bipyridine-6,6-dicarboxylic Acid. LCMS was used to verify the decay of DPA.

We acquired the following NMR-data from DPA and its photoproduct respectively: **2,6-Pyridinedicarboxylic acid (Dipicolinic acid, DPA) -** ^{1}H NMR (500 Mhz, D_2O, 296 K), δ/ppm: 8.63 (t, J= 7.8 Hz, 1H), 8.44 (d, J = 7.8 Hz, 2H). **2,2´- Bipyridine-6,6-dicarboxylic Acid -** ^{1}H NMR (500 Mhz, D2O, 296 K), δ/ppm: 8.70 (t, J = 7.9 Hz, 2H), 8.44 (d, J = 7.9 Hz, 2H), 8.15 (d, J = 7.9 Hz, 2H).

Parameter values for fitted curves in Figure 3B

Peak	Equation	Parameters
**782 cm^{-1}**	$y = A_1 + (A_2-A_1)/(1 + 10^{p(log_x0 - x)})$	$A_1 = 0.270$<br>$A_2 = 1.01$<br>$log_x0 = 26.3$<br>$p = -0.0441$
**1003 cm^{-1}**	$y = A_2 + (A_1-A_2)/(1 + (x/x_0)^p)$	$A_1 = 1.00$<br>$A_2 = 0.349$<br>$x_0 = 22.5$<br>$p = 1.65$
**1016 cm^{-1}**	$y = A_2 + (A_1-A_2)/(1 + (x/x_0)^p)$	$A_1 = 0.984$<br>$A_2 = -1.20 \times 10^{-4}$<br>$x_0 = 9.52$<br>$p = 3.67$
**1395 cm^{-1}**	$y = A_2 + (A_1-A_2)/(1 + (x/x_0)^p)$	$A_1 = 1.01$<br>$A_2 = 0.01$<br>$x_0 = 9.98$<br>$p = 3.55$
**1448 cm^{-1}**	$y = A_2 + (A_1-A_2)/(1 + (x/x_0)^p)$	$A_1 = 1.02$<br>$A_2 = 0.234$<br>$x_0 = 11.3$<br>$p = 1.96$
**1572 cm^{-1}**	$y = A_2 + (A_1-A_2)/(1 + (x/x_0)^p)$	$A_1 = 1.03$<br>$A_2 = 0.0624$<br>$x_0 = 11.0$<br>$p = 3.04$
**1670 cm^{-1}**	$y = A_1 {*} e^{(-x/t_1)} + y_0$	$y_0 = 1.01557$<br>$A_1 = -6.37436 \times 10^{-4}$<br>$t_1 = -9.87791$

Supplementary figures

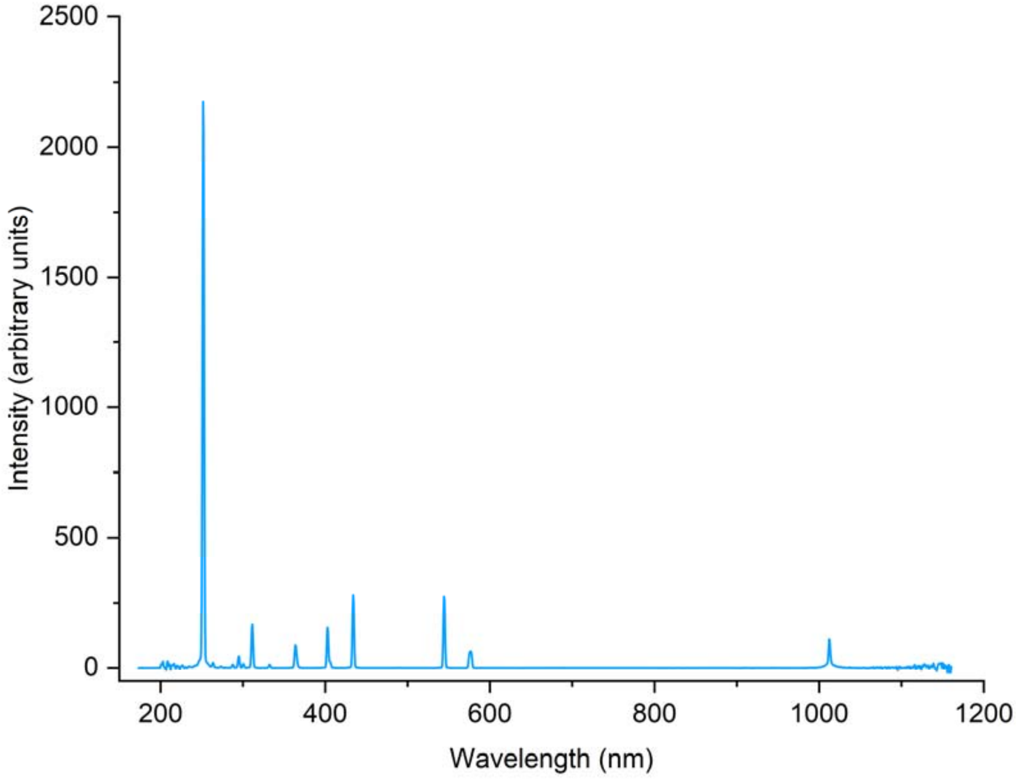

Figure S1: Emission profile of Hg-lamp used for decontamination. The most intense emission line is situated at 253.7 nm.

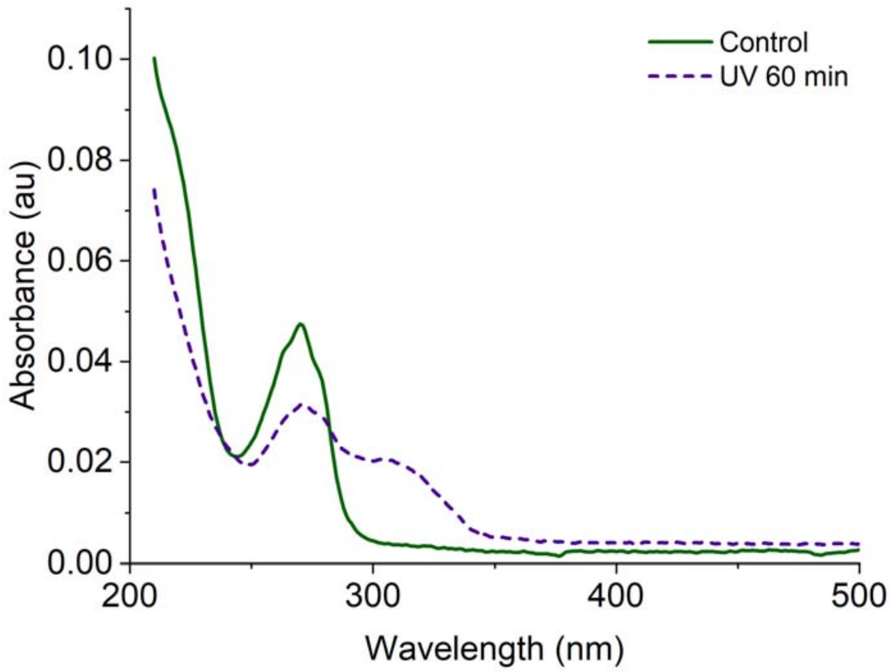

Figure S2: Absorbance of a 40 μM DPA solution before and after 60 minutes of UV-exposure.

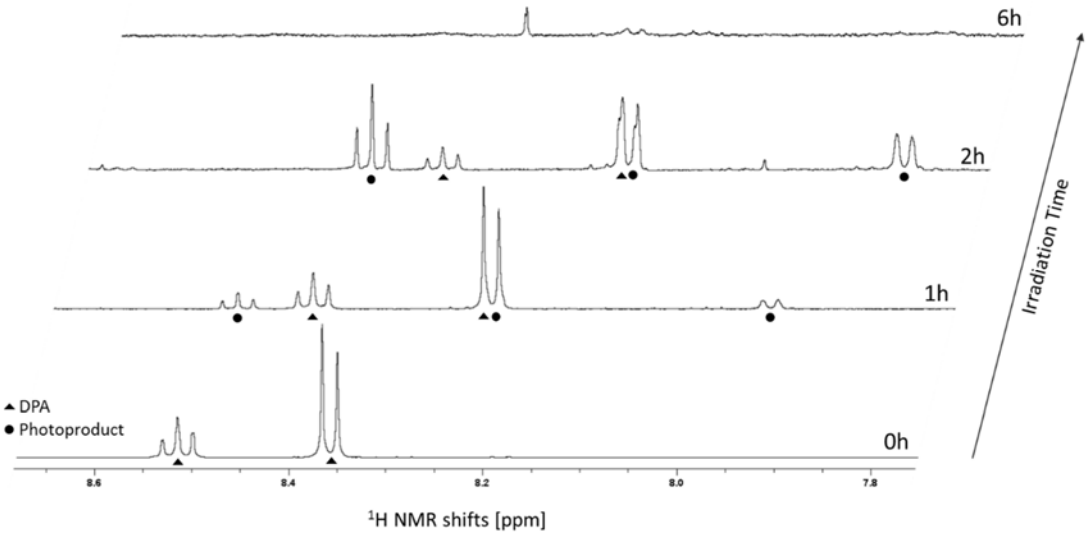

Figure S3: 1H NMR spectra obtained for a solution of 10 µM DPA in deuterium oxide after 1 h, 2 h and 6 h irradiation showing the decay of DPA (triangles) and the formation of the main photoproduct (dots). 1H NMR spectra of pre-irradiated DPA is added as reference (0 h).

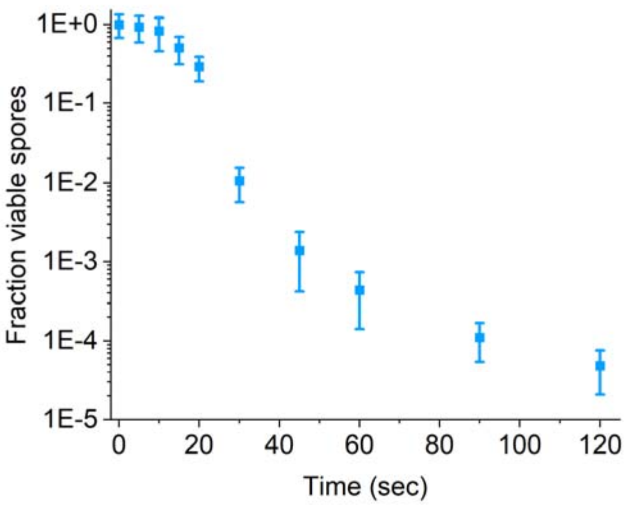

Figure S4: Viability of B. thuringiensis over two minutes of UV-exposure. Total amount of spores in suspension was estimated at $3x10^6$ spores.

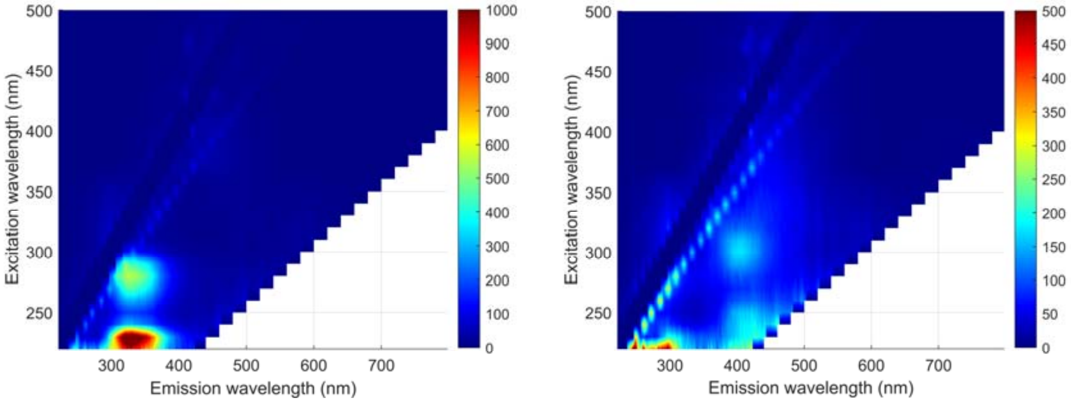

Figure S5: 2D fluorescence EEM for B. thuringiensis spores purified through Histodenz before (left) and after (right) 60 minutes of UV-exposure.

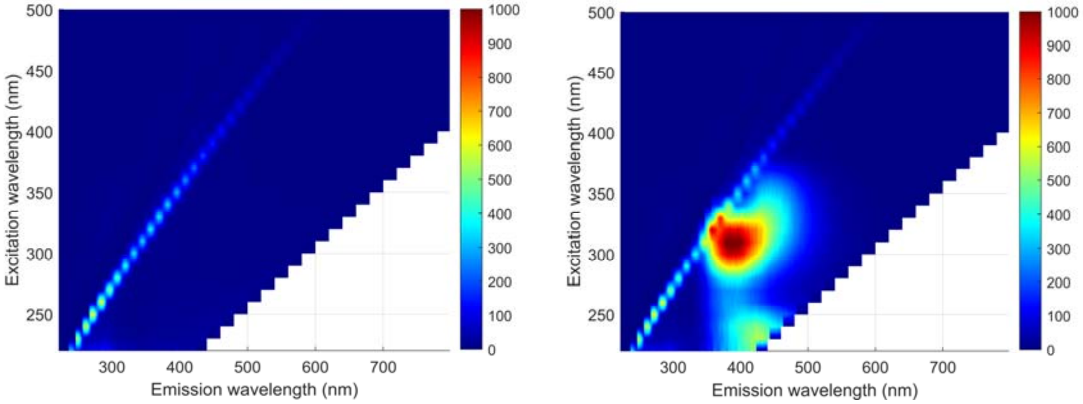

Figure S6: 2D fluorescence EEM of a 40 μM DPA solution before (left) and after (right) 60 minutes of UV-exposure.

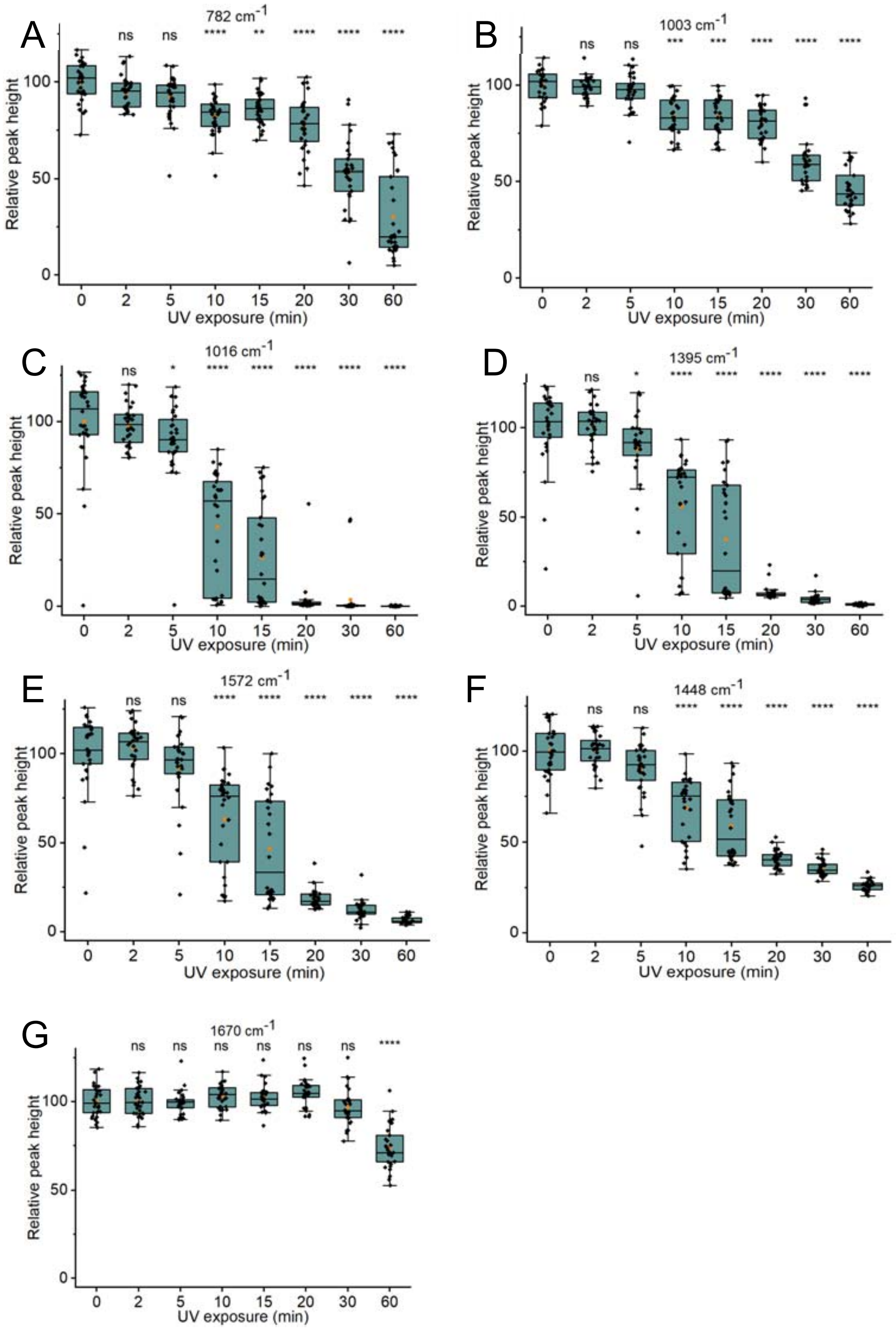

Figure S7: Measured individual Raman spectra intensities for selected peaks (n = 30 for each box). The box plots show the 1st and 3rd quartiles (box limits), median values (lines), 1.5·IQR (whiskers) and mean (orange dot). Statistically significant of the difference in distribution compared to the 0 min timepoint is shown above the boxes. The indicators are as follows; ns: not significant, "*": $p \leq 0.05$, "**": $p \leq 0.01$, "***": $p \leq 0.001$ and "****": $p \leq 0.0001$.

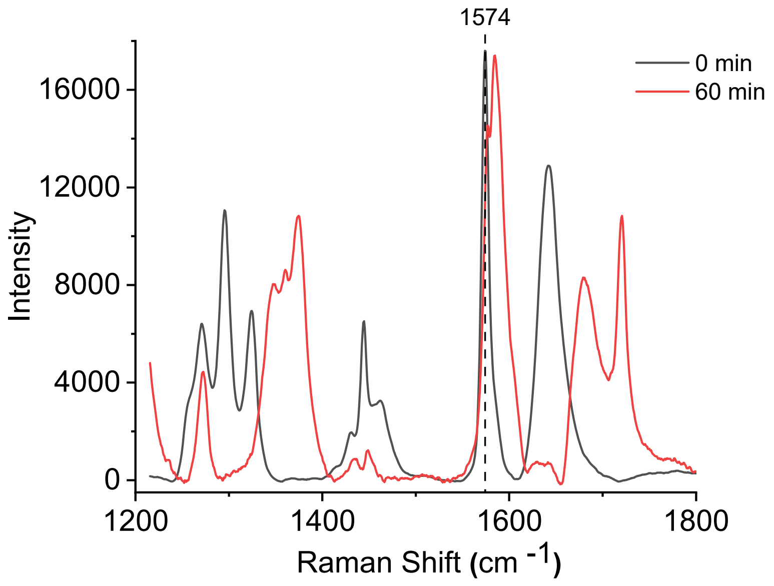

Figure S8: Representative Raman spectra of the DPA crystals of untreated DPA and after 60 min UV-exposure. The spectra differ greatly.

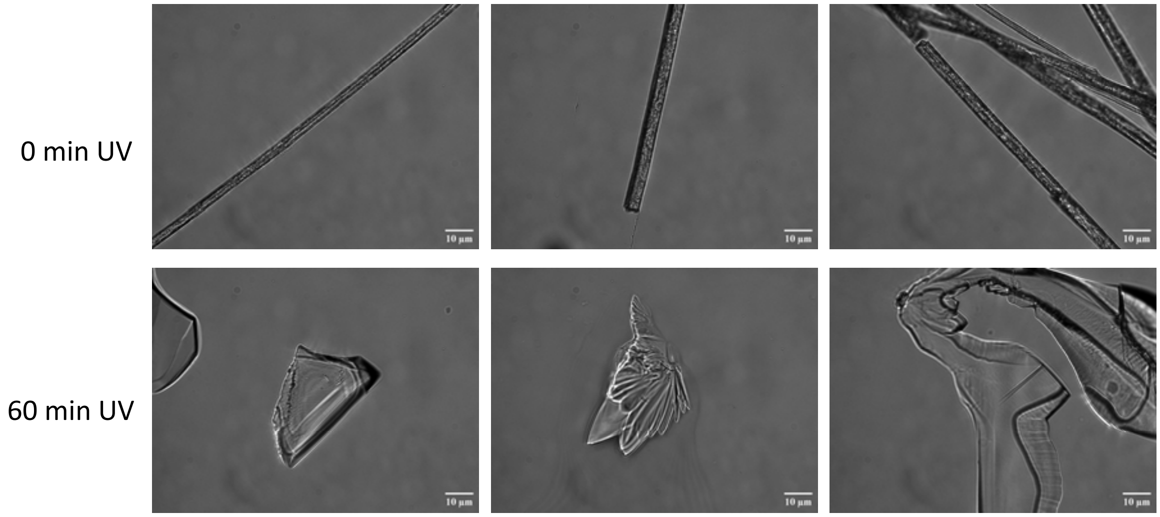

Figure S9: Differences in the crystal structure of 30 mM DPA dried on a glass slide. Untreated DPA forms long fibrous crystals. By contrast, UV-treated DPA no longer forms these fibres, instead forming glassy fragments of different sizes. The images are acquired using a 60× water immersion objective (UPlanSApo, Olympus) in brightfield mode of an inverted microscope (IX71, Olympus). The scale bar is 10 μm.

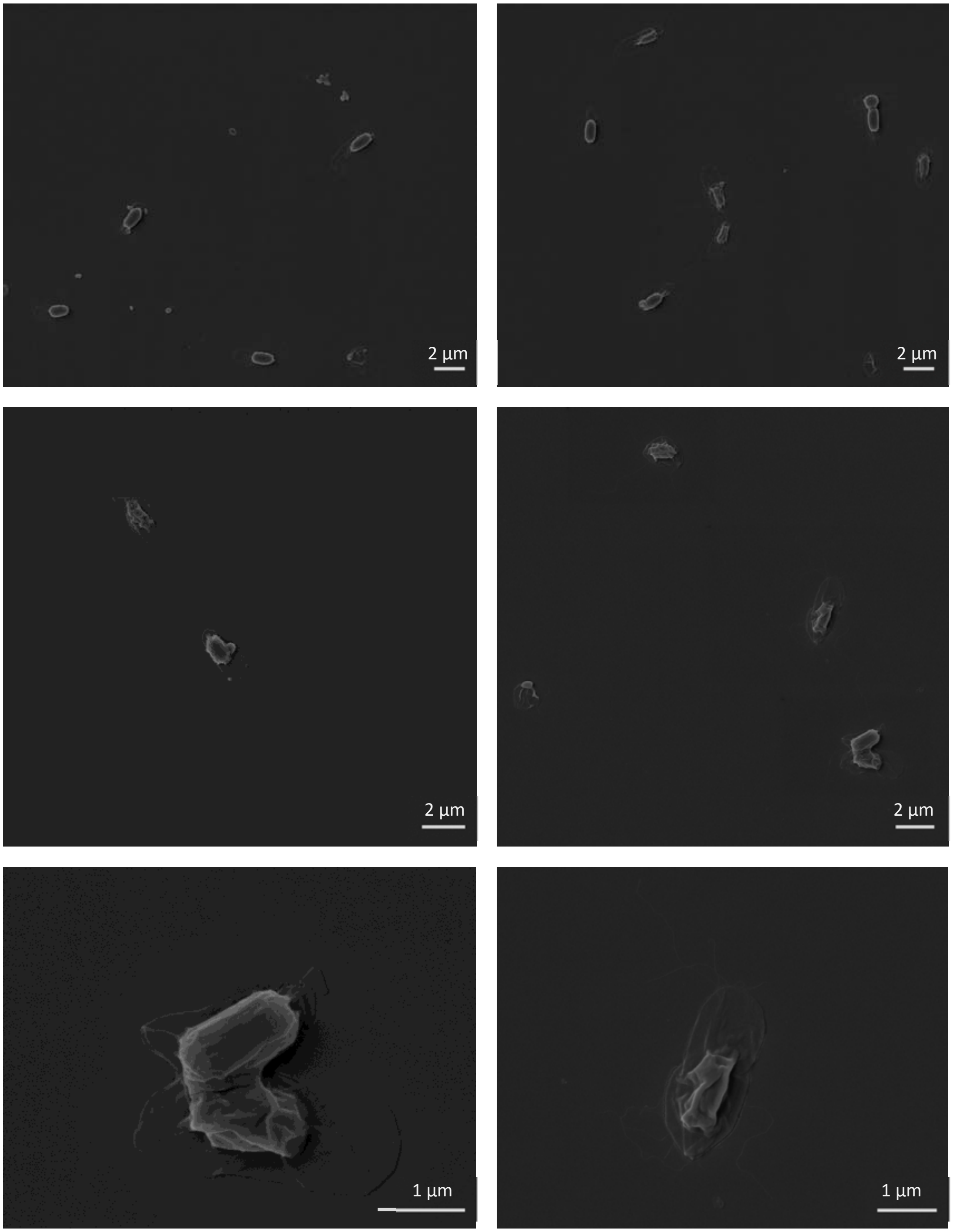
2 µm
2 µm
2 µm
2 µm
1 µm
1 µm

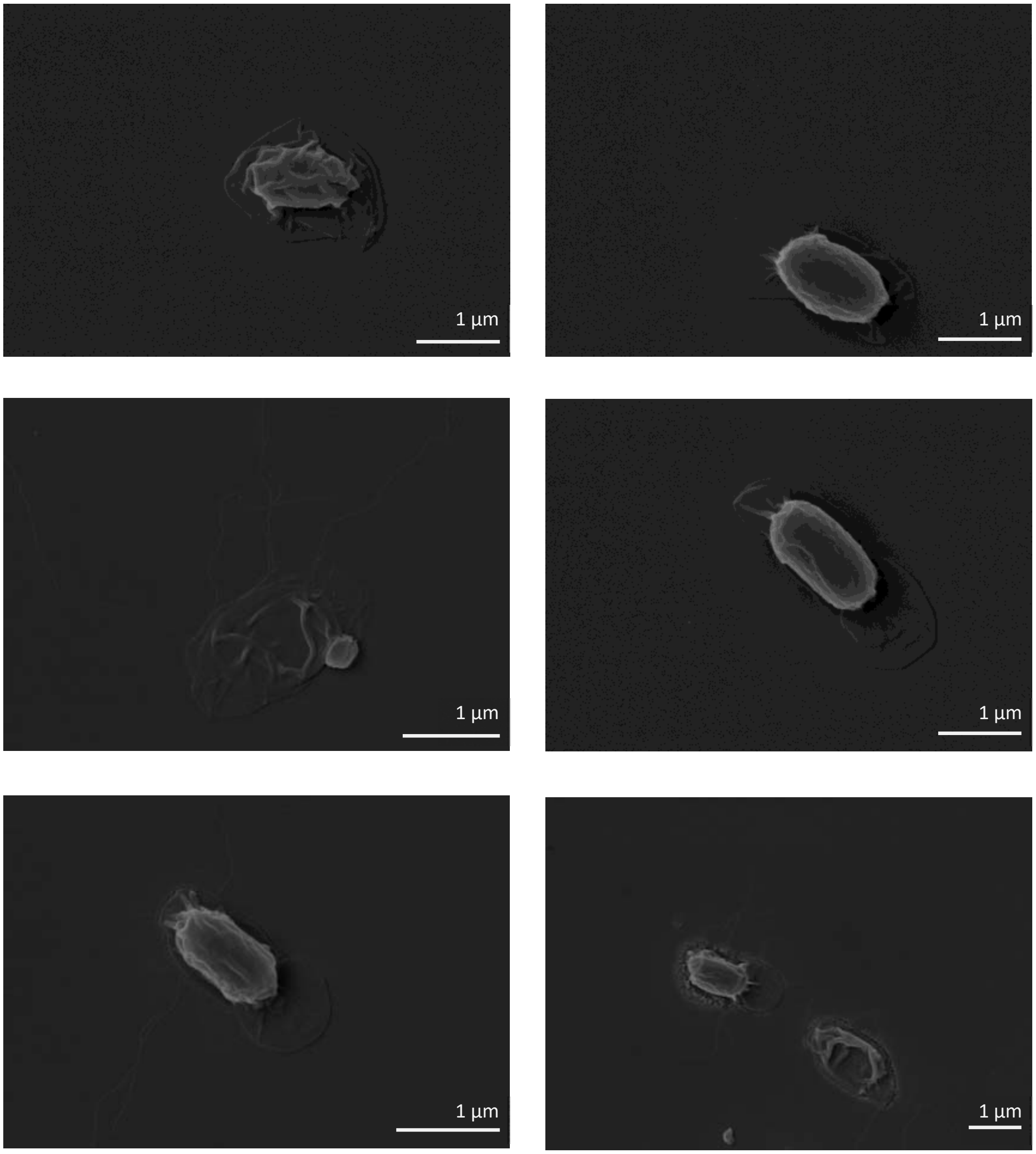

Figure S10: Additional SEM fields of untreated spores.

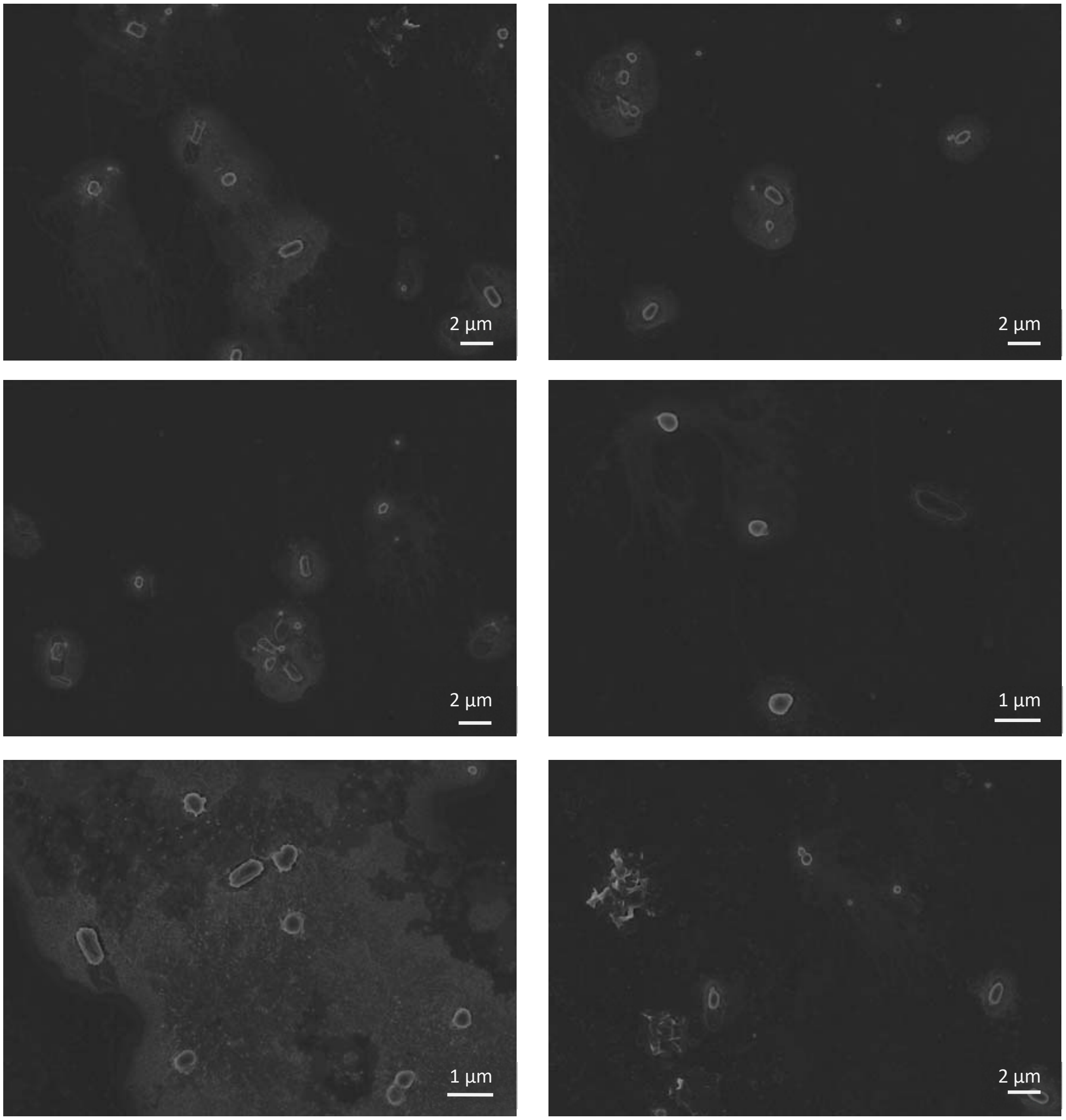
2 µm
2 µm
2 µm
1 µm
1 µm
2 µm

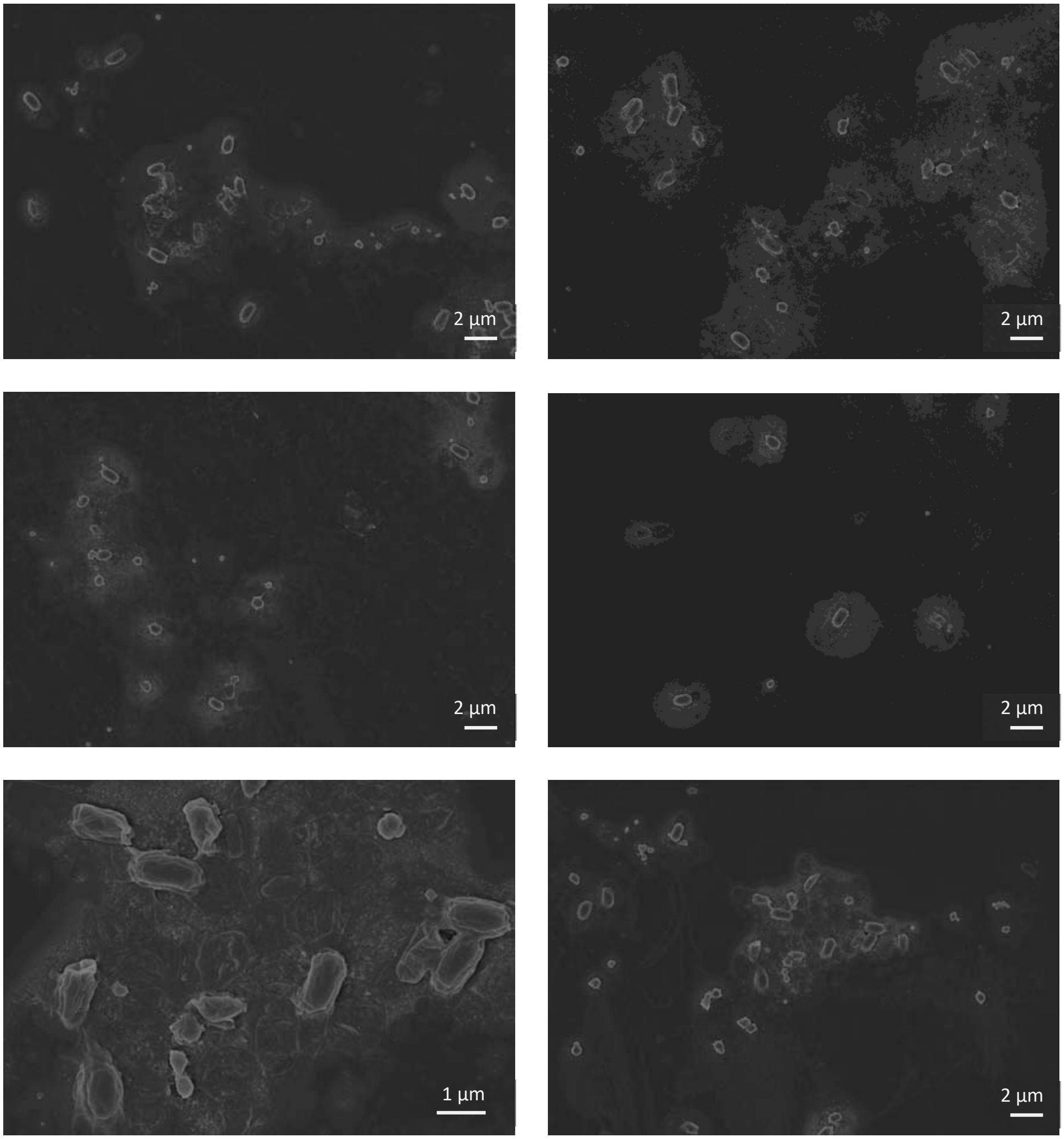

Figure S11: Additional SEM fields of spores illuminated for 60 min.

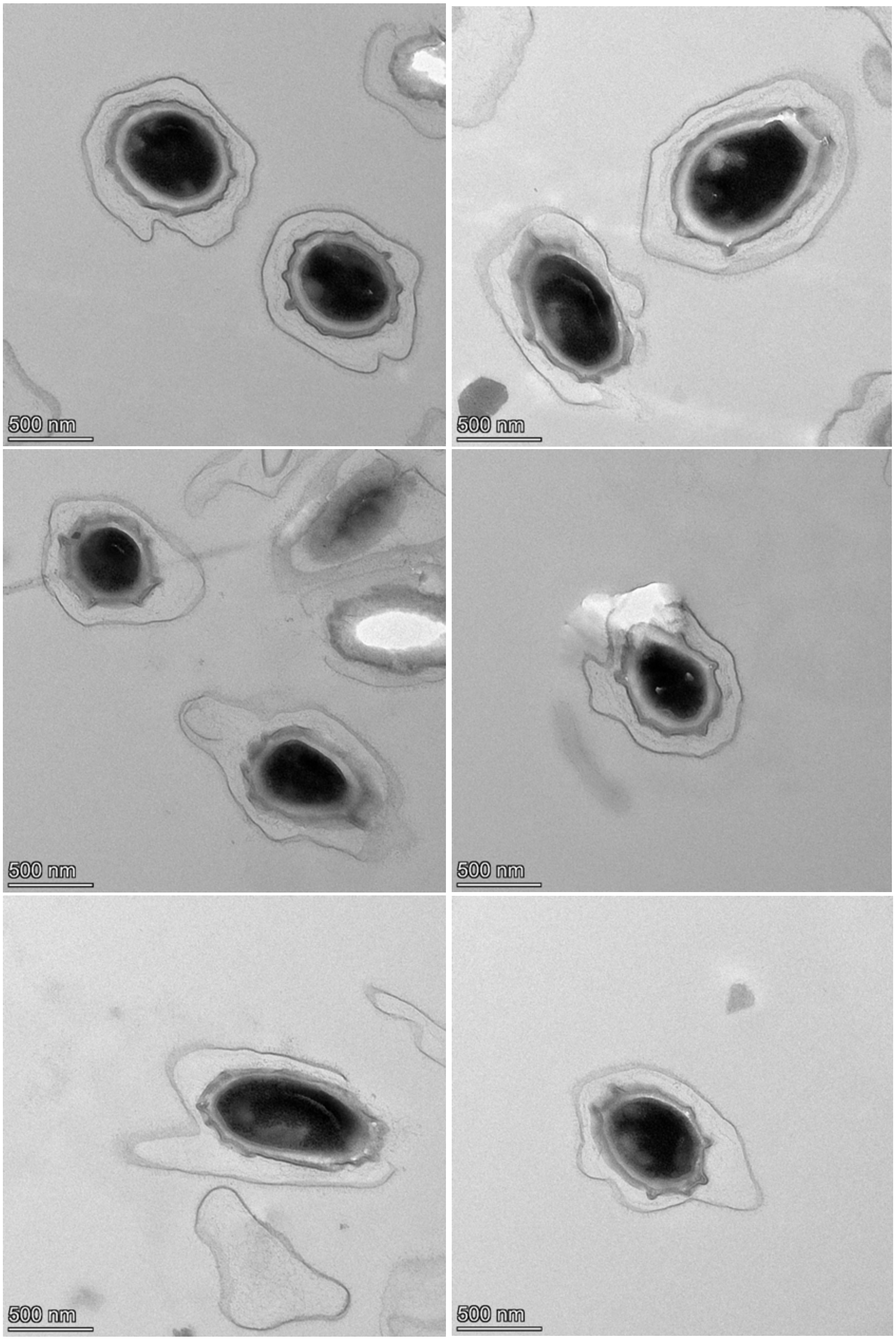
500 nm
500 nm
500 nm
500 nm
500 nm
500 nm

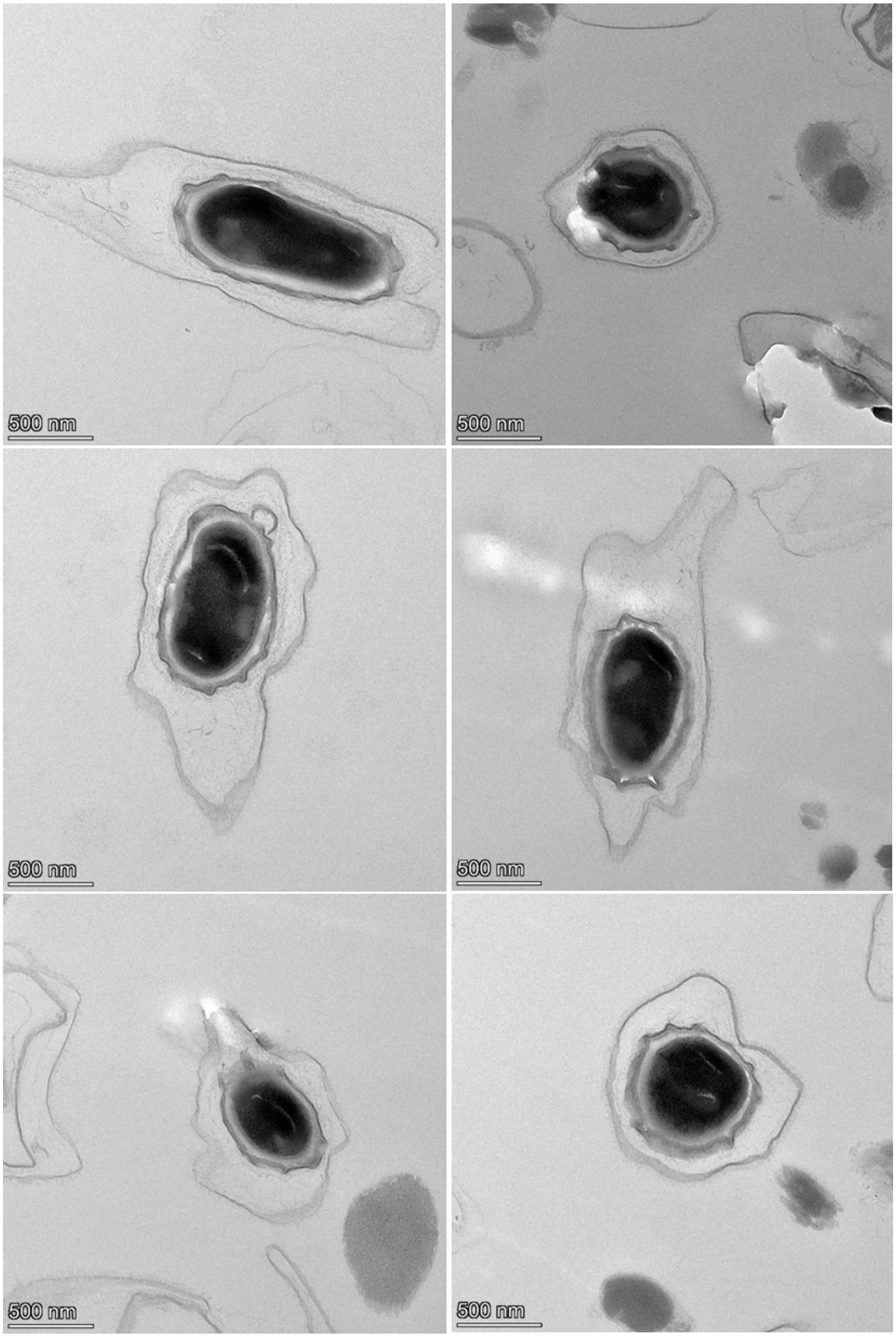

Figure S12: Additional TEMs of untreated spores.

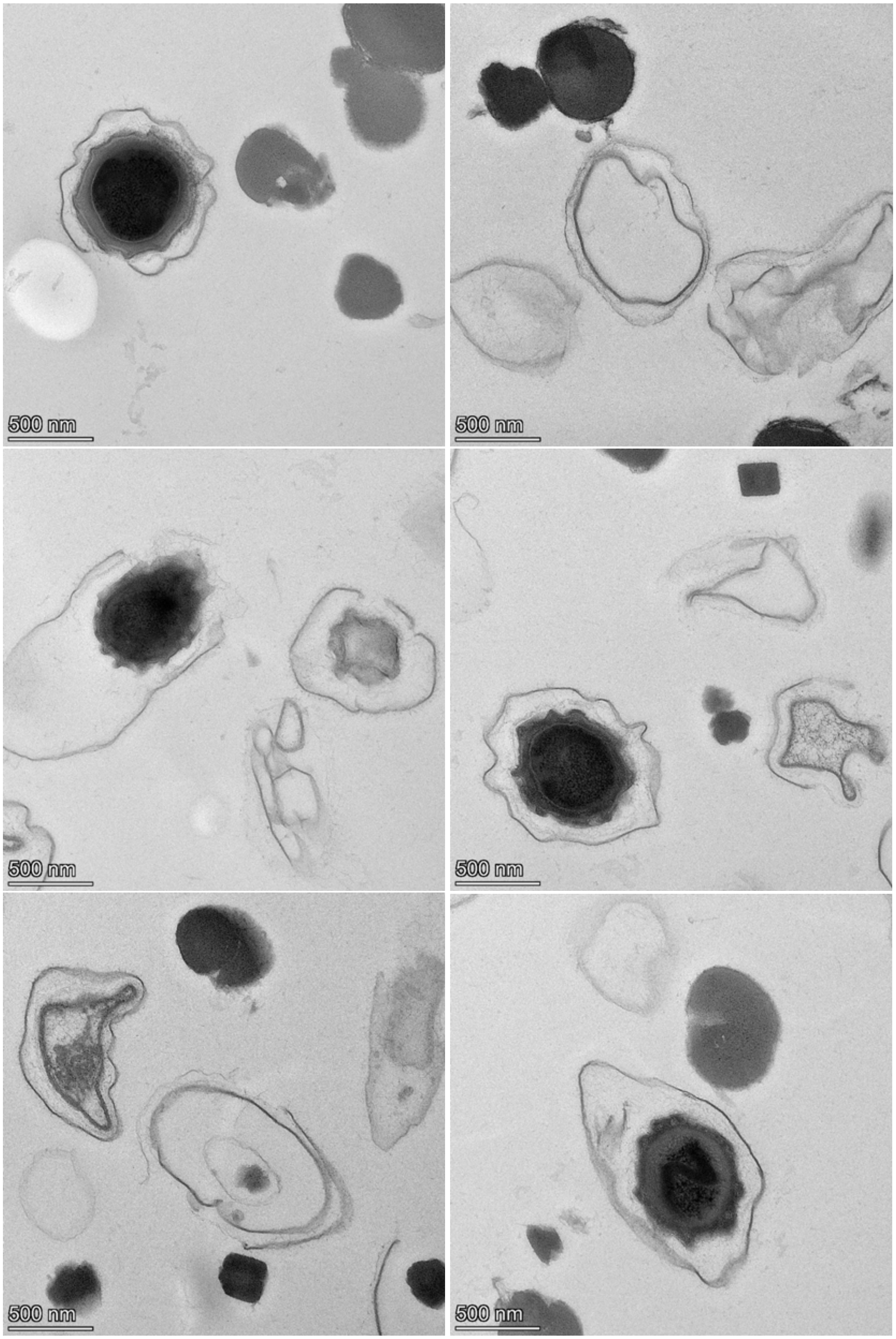
500 nm
500 nm
500 nm
500 nm
500 nm
500 nm

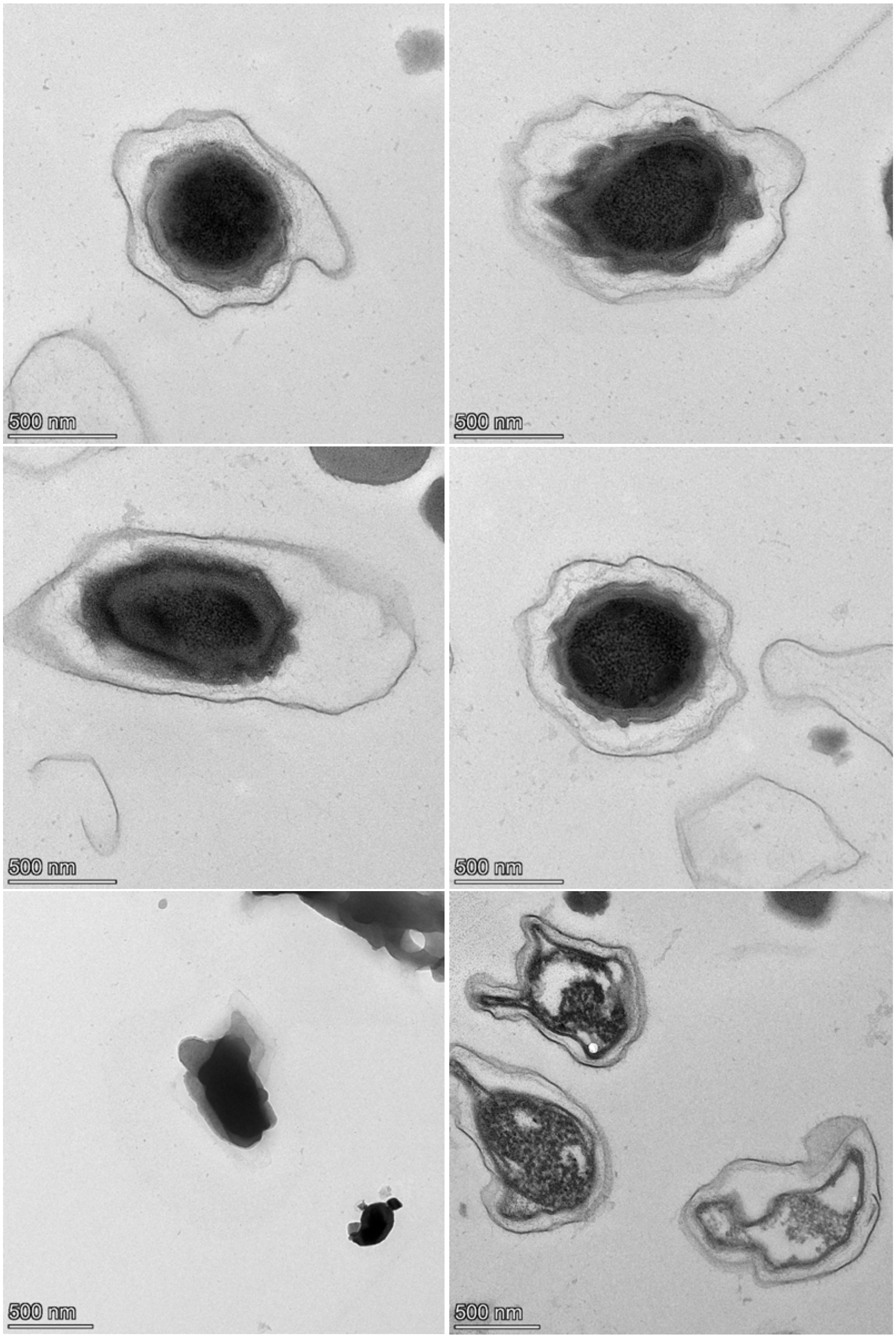

Figure S13: Additional TEMs of spores illuminated for 60 min.

References

[1] G. Nardi, M. Lineros-Rosa, F. Palumbo, M. A. Miranda, V. Lhiaubet- Vallet, Spectroscopic characterization of dipicolinic acid and its photoproducts as thymine photosensitizers, Spectrochimica Acta – Part A: Molecular and Biomolecular Spectroscopy 245 (2021) 118898.
